# Supplementary material for: Poly(ether-block-amide) copolymer membrane for CO2/N2 separation: the influence of the casting solution concentration on its morphology, thermal properties and gas separation performance
Source: R Soc Open Sci. 2019 Sep 4;6(9):190866. doi: 10.1098/rsos.190866 (PMC6774959; doi:10.1098/rsos.190866)
Supplement: Polyether-block-amide membrane: The Influence of casting solution concentration on its morphology, thermal properties and gas separation performance [file rsos190866supp1.pdf]

Polyether-*block*-amide copolymer membrane for CO_2/N_2 separation: The Influence of casting solution concentration on its morphology, thermal properties and gas separation performance

Lidia Martínez-Izquierdo, Magdalena Malankowska,* Javier Sánchez-Laínez, Carlos Téllez and Joaquín Coronas*

Theoretical background

d-spacing and crystallinity (X_c) calculations

In order to study the morphology of the membranes, the molecular distances between polymer chains (*d*-spacing) and the crystallinity can be estimated using different techniques such as X-ray diffraction (XRD) or differential scanning calorimetry (DSC). In this study, the *d*-spacing was calculated applying Bragg's law to the data collected from XRD, Eq. (S1):

$$n \cdot \lambda = 2 \cdot d_{sp} \cdot \sin\theta \qquad \text{(S1)}$$

where n is a positive integer, λ is the wavelength (λ=0.154 nm), d_{sp} is the *d*-spacing (nm) and θ is the angle of incidence. The polymer crystallinity was estimated with the data obtained by DSC, applying the following equation, Eq. (S2):

$$X_c\ (\%) = \frac{\Delta H_m}{\Delta H_m^0} \cdot 100 \qquad \text{(S2)}$$

where X_c is the crystallinity in %, ΔH_m is the melting enthalpy ($J \cdot g^{-1}$), calculated integrating the area under the melting peak, and ΔH_m^0 ($J \cdot g^{-1}$) is the melting enthalpy of the polymer 100 % crystalline. In the case of PEBA, the crystallinity of both segments has been determined using 147 $J \cdot g^{-1}$ and 230 $J \cdot g^{-1}$ as the enthalpy of the 100 % crystalline polyethylene oxide (PEO) and polyamide (PA), respectively.[1]

Apparent activation energy (Ea)

In chemistry, the minimum amount of energy required by a chemical reaction to occur is called activation energy. Changes in temperature or pressure also imply changes in other molecular properties such as chemical velocity, gas permeability, gas diffusivity and solubility, relative crystallinity, etc.[2, 3] usually following an Arrhenius equation, Eq. (S3):

$$k(T) = k_0 \cdot \exp\left(-\frac{E_a}{R \cdot T}\right) \qquad \text{(S3)}$$

where k(T) is a rate constant, k_0 is the pre-exponential factor, E_a is the apparent activation energy in $J \cdot mol^{-1}$, R the ideal gas constant 8.314 J $mol^{-1} \cdot K^{-1}$ and T is the temperature in K.

Very often, the knowledge of the kinetic model becomes a problem in a kinetic study. For this reason, integral methods, which do not assume any kinetic model, are widely employed for the estimation of the apparent activation energy.[4-6] In the present study, two different integral methods have been used to calculate the apparent activation energy of the membranes thermal degradation. These are the Kissinger and Ozawa Integral methods.

Kissinger,[7] developed a procedure to calculate the activation energy collecting data of thermal degradation (differential thermal analysis) at different heating rates, demonstrating that the increment of the heating rate led to a displacement of the maximum degradation peak to higher temperatures, Eq. (S4):

$$\ln\frac{\beta}{T_m^2} = \ln\frac{k_0 \cdot R}{E_a} - \frac{E_a}{R \cdot T_m} \qquad \text{(S4)}$$

Ozawa,[8] suggested a method to study the process of nucleation and growth in non-isothermal cold crystallization. Such technique was based on the estimation of the modified activation energy with data obtained from tests conducted by cooling poly(ethylene-terephthalate) at constant heating rates, using DSC, Eq. (S5):[9]

$$\ln\beta = \ln\frac{0.00484 \cdot k_0 \cdot E_a}{G(\alpha) \cdot R} - 1.0516 \cdot \frac{E_a}{R \cdot T_m} \quad (S5)$$

In both equations (S4) and (S5), β is the heating rate ($K \cdot min^{-1}$), T_m is the temperature of the maximum weight loss (K), E_a is the degradation apparent activation energy ($J \cdot mol^{-1}$), k_0 is the pre-exponential factor in s^{-1} and R is the ideal gas constant in $J \cdot mol^{-1} \cdot K^{-1}$. In Ozawa's equation, G(α) is a constant value.

Results

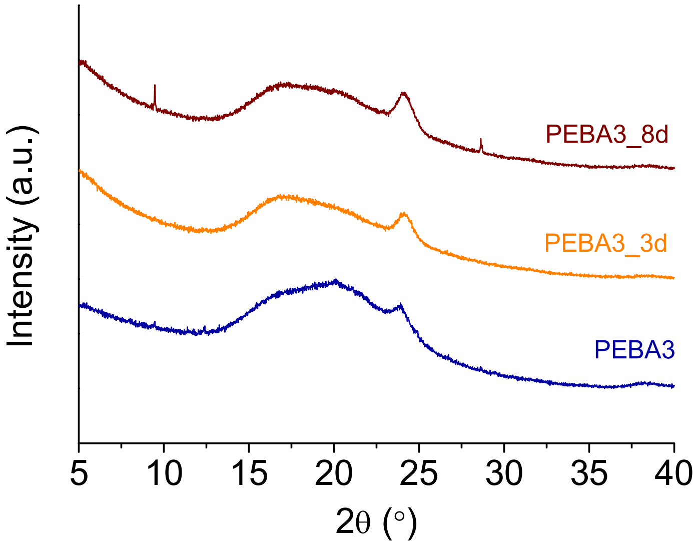

Figure S1 XRD patterns of PEBA membranes with and without thermal treatment (3 and 8 days at 150 ºC).

Experimental X-ray diffraction (XRD) of PEBA3 membranes with and without thermal treatment at 150 ºC for different periods of time (3 and 8 days) suggests that the crystallinity is affected by the time that the membrane is at this conditions. In fact, the crystallinity is higher when the time that the sample is under this conditions increase. Despite the higher crystallinity, the sample changed its colour when subjected to thermal treatment, indicating that a partial degradation has taken place during the heating.

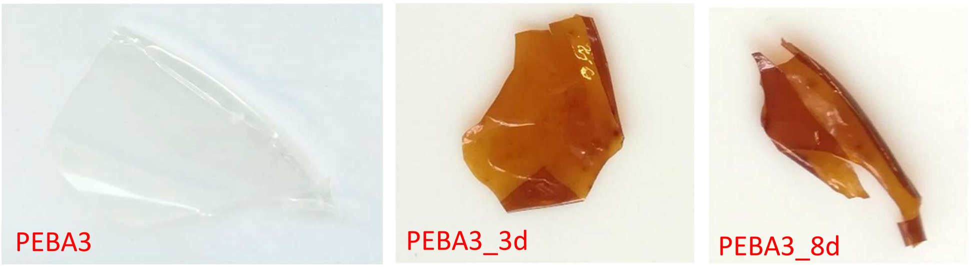

Figure S2 Pictures of the membranes without (PEBA3) and with thermal treatment after 3 (PEBA3_3d) and 8 (PEBA3_8d) days at 150 ºC.

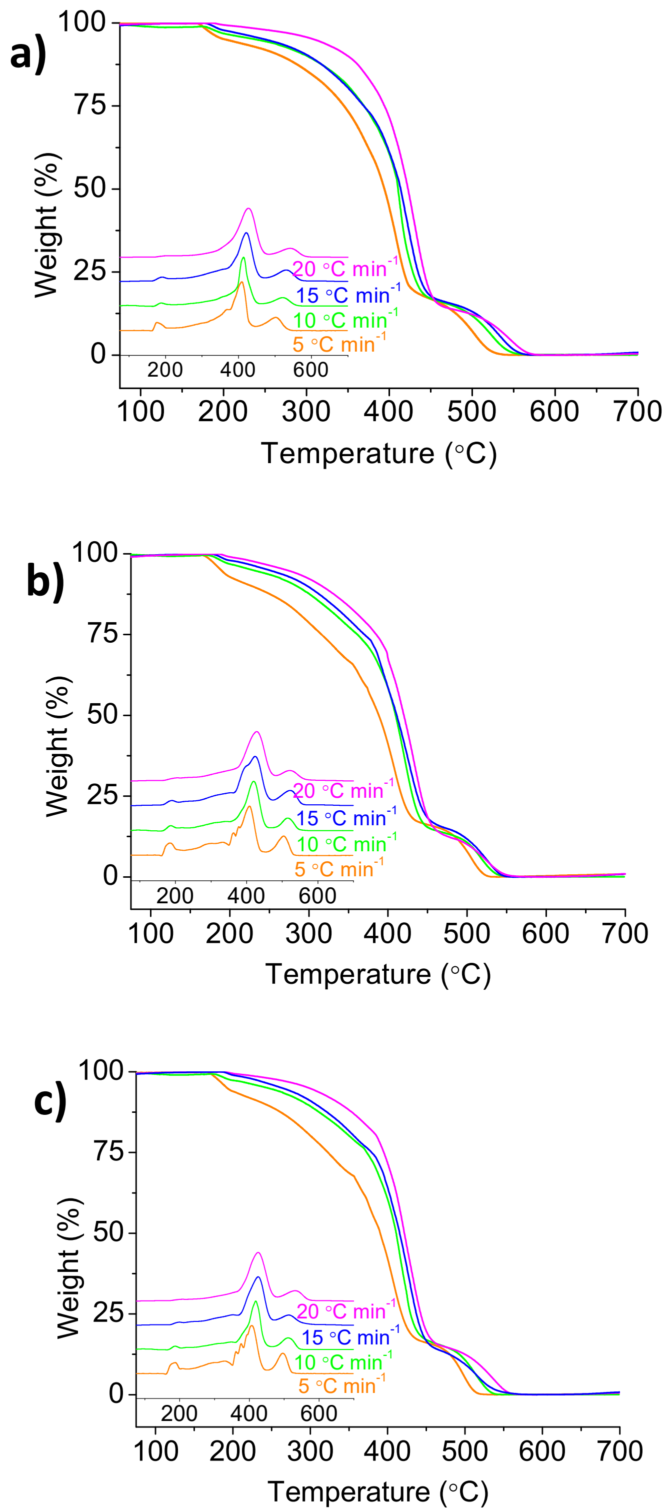

Figure S3 TGA and DTG curves of PEBA1 (a), PEBA3 (b) and PEBA5 (c) at different heating rates (5, 10, 15 and 20 °C min^{-1}), oxidized in air atmosphere.

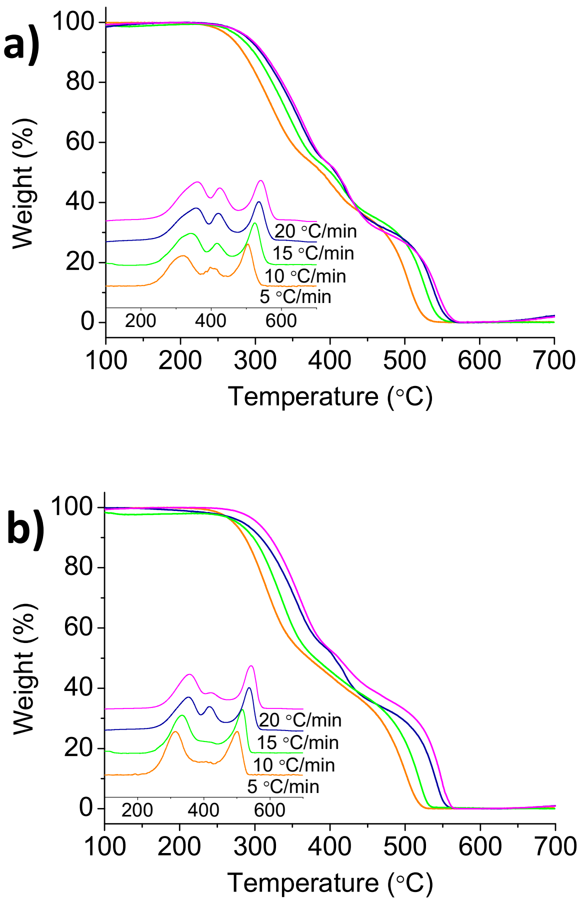

Figure S4 TGA and DTG curves of PEBA3 treated at 150 ºC during 3 days (a) and 8 days (b), at different heating rates (5, 10, 15 and 20 ^{o}C min^{-1}).

With the data obtained by TGA, we have been able to calculate the apparent activation energy of these treated membranes and compare them with that of the PEBA3 without post-treatment. The values obtained show an important decrease in the activation energy, which corroborates the partial degradation of the samples. This fact also implies that the membranes have lost thermal stability.

Table S1 Apparent activation energies calculated with the Kissinger and Ozawa methods for the membranes treated at 150 ^{o}C.

	T_{max}(1) (^{o}C)	T_{max}(2) (^{o}C)	E_a(a) (kJ mol^{-1})	E_a(b) (kJ mol^{-1})
PEBA3	420	515	263	261
PEBA3_3d	342 - 417	523	180	184
PEBA3_8d	335 - 417	518	148	153

(1) Thermal decomposition at a heating rate of 10 ^{o}C min^{-1} (for PEBA3_3d and PEBA3_8d, the two values of temperature correspond to the two peaks that appear in this decomposition step), (2) oxidation step at a heating rate of 10 ^{o}C min^{-1}, (a) calculated with Kissinger equation and (b) with Ozawa method

References

1. S. J. Kim, H. Jeon, D. J. Kim and J. H. Kim, *ChemSusChem*, 2015, **8**, 3783-3792.
2. A. Guo, Y. Ban, K. Yang and W. Yang, *Journal of Membrane Science*, 2018, **562**, 76-84.
3. R. M. R. Wellen and E. L. Canedo, *Polymer Testing*, 2014, **40**, 33-38.
4. P. Rajeshwari, *Journal of Thermal Analysis and Calorimetry*, 2015, **123**, 1523-1544.
5. A. Y. T. Snegirev, V. A.; Stepanov, V. V.; Harris, J. N., *Thermochimica Acta*, 2012, **548**, 17-26.
6. X. Zuo, H. Shao, D. Zhang, Z. Hao and J. Guo, *Polymer Degradation and Stability*, 2013, **98**, 2774-2783.
7. H. E. Kissinger, *Journal of Research of the National Bureau of Standards*, 1956, **57**, 217-221.
8. N. Coburn, P. Douglas, D. Kaya, J. Gupta and T. McNally, *Advanced Industrial and Engineering Polymer Research*, 2018, **1**, 99-110.
9. H. Cheng, Q. Liu, P. Xu and R. Hao, *Journal of Solid State Chemistry*, 2018, **268**, 36-44.
